# Supplementary material for: Are carbon emissions trading and green financial instruments synergistic? -Comprehensive quantitative research based on content analysis
Source: PLoS One. 2024 Mar 7;19(3):e0298601. doi: 10.1371/journal.pone.0298601 (PMC10919601; doi:10.1371/journal.pone.0298601)
Supplement: S1 Data — (ZIP) [file pone.0298601.s001.zip › Data/地级市Z_F工作报告.zip!/╒π╜¡/╝╬╨╦╩╨/└┤╫╘╣1⁄2╓┌║┼í╛┬φ┐╦╩2╛▌═°í┐_2017.docx]

各位代表：

现在，我代表市人民政府向大会报告工作，请予审议， 并请市政协委员和其他列席人员提出意见。

一、2016 年主要工作和过去五年发展成效

2016 年是实施“十三五”规划的开局之年。面对错综复杂的外部环境和繁重的改革发展稳定任务，在省委、省政府和中共嘉兴市委的坚强领导下，市政府全面落实党的十八大和十八届三中、四中、五中、六中全会精神，深入贯彻习近平总书记系列重要讲话精神，统筹推进“五位一体”总体布局和协调推进“四个全面”战略布局，主动适应经济发展新

常态，坚持稳中求进工作总基调，统筹做好经济社会发展各项工作，较好地完成了市七届人大七次会议确定的各项目标任务。

——经济运行稳中向好。全市地区生产总值达到 3760

亿元，增长 7%，其中市本级增长 7.5%。实现一般公共预算收入 387.9 亿元，增长 10.4%，其中市本级增长 14.1%。固定资产投资增长 11%，其中“四个重大”投资增长 17.4%。社会消费品零售总额达到 1638.5 亿元，增长 10.8%。农林牧渔业增加值增速扭转了负增长局面，实现转负为正。规上工业企业利润增长 25.9%，单位生产总值能耗下降 2.6%。经济发展的质量和效益明显提高。

——扩大开放深入推进。对外贸易实现逆势回升，进出

口总额增速列全省第二，其中出口增长 9%，增速列全省第一。招大引强选优成效明显，实际利用外资再创新高，总量列全省第三。引进世界 500 强、国际行业领先企业和 1 亿美元以

上重大项目 30 个，总投资超 200 亿元的德国宝沃汽车项目落户我市。浙商回归考核连续五年列全省同档城市第一。

——发展新动能不断增强。新兴产业快速增长，规上工

业增加值占到全部工业的 89.4%，装备制造业、高新技术产业、战略性新兴产业占规上工业增加值比重分别达到 27.1%、

45%和 37.2%，新产品产值率提高到 40%，高新技术产品出口增长 35.3%。服务业增加值占地区生产总值比重提高到

45.3%。电子信息制造业产值、网络零售额均突破千亿大关。

大众创业、万众创新蓬勃开展，全市新增市场主体 8 万户， 增长 25.2%，其中新登记注册企业 2.1 万户，增长 30.8%。新动能正在撑起发展新天地。

——基础设施建设持续推进。完成基础设施投资 620 亿

元，增长 17.7%。杭州湾跨海大桥北接线二期、杭州至海宁城际铁路、京杭运河航道改造等一批重大交通项目开工建 设。国家海绵城市建设试点稳步推进。平湖塘延伸拓浚、杭嘉湖扩大南排工程进展顺利。“光网城市”“无线城市”建设成效显著，实现百兆家庭光纤宽带、4G 网络、重点公共场所免费 WIFI“三个全覆盖”。

——人民生活进一步改善。城乡居民人均可支配收入分

别达到 48926 元和 28997 元，收入差距比缩小到 1.687︰1。坚持将有限的资金更多地用于保障和改善民生，财政民生支出占一般公共预算支出比重首次达到 80%，新开工建设保障性住房 3 万套，发放各类救助金 2.4 亿元。城乡居民生活水平有了新提高。

回顾过去一年，形势严峻复杂，成绩来之不易。这些成绩，是在世界经济和贸易增速七年来最低、国内结构性矛盾突出、经济下行压力加大的情况下取得的。面对经济工作中两难甚至多难的问题和困境，我们始终坚持稳中求进、科学发展，咬定青山不放松，稳扎稳打、有效应对，实现了“十三五”良好开局。实践再次证明，任何艰难险阻都挡不住嘉兴人民坚毅前行的步伐。面对未来，我们有信心、有决心、

有条件，更有办法不断创造新的更大辉煌！ 一年来，我们主要做了以下工作。

一是着力抓好“三去一降一补”。大力推进去产能。开展“退低进高”“退散进集”行动，整治提升“低散弱”企业 2929 家，处置“僵尸企业”83 家。房地产去库存取得积极成效。制定《促进商贸综合体和专业市场规范发展的若干意见》，全市商品住宅去库存完成省下达目标任务。加强金 融风险防范。规上工业企业资产负债率下降到 54.4%，银行金融机构不良贷款率保持全省最低。出台《降低企业成本优化发展环境的政策意见》，减免企业税费 153 亿元。加快补齐科技创新、低收入群体增收等短板，全社会研究与试验发展经费支出增长 11.8%，低收入农户人均可支配收入增长

18.1%。

二是推动重大改革举措落地。全面深化“四张清单一张网”改革，开展相对集中行政许可权、核准目录外企业投资项目不再审批等改革试点，建成统一政务咨询投诉举报平 台，实现市、县、镇三级权力清单和责任清单全覆盖。深入实施资源要素差别化配置改革，完善以亩产效益论英雄的综合评价机制，建成市县一体的公共资源交易平台。开展国资公司降负债成本、降应收款项、降库存、降管理成本专项行动。稳步推进农村“三权”改革。深化户籍制度改革，全面实施新型居住证制度试点。加快推进省港产城统筹发展试验区建设，嘉兴港口岸实现全域对外开放。

三是强化科技创新引领。深入实施创新驱动发展战略， 成功获批省全面创新改革试验区，秀洲国家高新区正式挂牌，嘉兴科技城扩容升级迈出实质性步伐。统筹抓好增加科技投入、加强研究开发、推进成果转化等工作，新增发明专利授权 1654 件，新认定高新技术企业 184 家，14 家企业跻身省高新技术企业“百强榜”，全国首家专业科技保险公司落户我市，实现国家级科技企业孵化器县（市、区）全覆盖。提升扩大“创新嘉兴﹒精英引领计划”成果效应，截至 2016

年底，累计设立院士专家工作站 58 个，“国千”“省千”人

才分别达到 161 名和 136 名。

四是加快产业转型升级。制定实施《中国制造 2025 嘉

兴行动纲要》，积极推进“四换三名”，完成工业技改投资 1023 亿元，新增“浙江名牌”32 个、上市公司 4 家、新三板挂牌企业 44 家。我市被工信部评为全国工业稳增长和转型升级成效明显市。乌镇国家互联网创新发展试验区建设进展顺 利，成功承办第三届世界互联网大会·乌镇峰会。实施加快发展现代服务业行动计划，完成服务业“百项千亿”投资 287 亿元。4 个小镇入选第二批省级特色小镇。西塘古镇成功创建国家 5A 级景区。深化农业“两区”建设，1 个农业产业集聚区、2 个特色农业强镇列入省首批“一区一镇”试点。

五是全面推进新型城镇化建设。深化“多规合一”试点， 完成市域总体规划修编。在全省率先启动小城镇环境综合整治，部署实施镇村环境整治提升三年行动计划，新增省级小

城市培育试点镇 3 个。深入推进“三改一拆”，完成“三改”

面积 3476 万平方米，拆除违法建筑 1269 万平方米。城市有机更新取得新进展，优化子城片区规划与城市设计，文生修道院、杉青闸片区全面完成房屋征收。加快市域千里绿道建设，截至 2016 年底，全市建成绿道 902 公里，其中市区 501 公里。科学开展城市交通治堵，中心城区新建改建商务大道、万国路等 8 条城市道路，新增公交专用道 10 公里，中山路与越秀路人行天桥主体工程完工。

六是深入开展治水治气。深化“五水共治”。大力推进 城乡生活污水治理、工业企业污水全入网、饮用水水源地达标提升等工程。新建城镇污水管网 391 公里，完成河道清淤

1358 万立方米。全市跨行政区域河流交接断面水质考核保持优秀。市域外配水工程（杭州方向）确定线路并完成初步设计。深化“五气共治”。实行全域露天秸秆、废弃物禁烧， 推进 12 个主要行业、28 家燃煤热电企业、59 家年耗煤万吨以上企业工业废气整治，淘汰小锅炉 2101 台、黄标车 1760 辆。市区空气质量优良率达到 74.3%，PM2.5 平均浓度 44 微克/立方米，下降 17%。加大环境执法力度，查处环境违法案件 1141 起。

七是协调发展各项民生事业。统筹做好重点人群就业工作，新增城镇就业 10.4 万人。构建社会“大救助”体系，在全省率先建立支出型贫困家庭救助制度。以东部地区第一的成绩成功创建国家公共文化服务体系示范区，农村“文化礼

堂”覆盖率全省第一，子城遗址发掘取得重要成果。深化国家特殊教育改革实验区、职业教育现代学徒制试点等改革， 南洋职业技术学院新校区、浙江大学国际联合学院（海宁校区）建成启用。积极接轨上海高端优质医疗资源，与解放军第二军医大学“一校三院”开展全方位紧密型合作，让嘉兴老百姓在家门口就能享受到“接轨上海”的医疗服务。健全完善“双下沉、两提升”、分级诊疗、家庭责任医生签约“三位一体”医疗服务模式，推行便捷支付诊间结算，顺利通过国家卫生城市复评。大力开展全民健身运动，承办省第二届女子体育节。强化安全生产、食品药品安全监管，组织开展消防安全大排查大整治行动，安全生产事故起数、死亡人数分别下降 14%和 9.3%。健全立体化社会治安防控体系，圆满完成平安护航 G20 杭州峰会、第三届世界互联网大会·乌镇峰会安保维稳任务。加强国防建设、民兵预备役和人民防空工作。实现创建全国双拥模范城“三连冠”。

八是切实加强依法行政。遵照授予有据、行使有规、监督有效的原则，自觉接受市人大及其常委会监督和市政协民主监督，办理市人大代表建议 238 件、市政协提案 352 件。

建立市政府常务会议学法制度，集中学习法律法规规章 14

部。清理政府规范性文件 550 件，办理行政复议案件 77 件。严格“三公”经费支出管理，实施公务用车制度改革。扎实开展“两学一做”学习教育，政府系统廉政建设全面加强。

2016 年是本届政府任期的最后一年。五年来，市政府坚

持把发展作为第一要务，紧紧团结和依靠全市人民，克难奋进、开拓创新，实现了本届政府各项任务的圆满收官，全市经济社会发展迈上了一个新台阶。五年间，经济在转型升级中量质齐升。地区生产总值、一般公共预算收入年均分别增长 7.9%和 11.4%，人均地区生产总值由 6 万元提高到 8.2 万元。全社会研究与试验发展经费支出占地区生产总值比重从

2.2%提高到 2.8%，三次产业结构由 5.3︰56.7︰38 调整为

3.8︰50.9︰45.3。五年间，改革在先行先试中砥砺奋进。先后获批新型城镇化、“多规合一”、“医养结合”等 92 项国家、省改革试点，在全省率先实施资源要素差别化配置、行政审批层级一体化、综合行政执法等改革，分布式光伏发电模式在全国得到推广，产业升级、农业农村、社会治理、政府职能转变等重点领域改革全面推进，初步形成了具有嘉兴特色的改革体系。五年间，城乡在统筹发展中走在前列。“1640”

“四百一千”空间布局进一步完善，累计完成土地复垦 5.3 万亩，改造集聚农房 7.5 万户，常住人口城镇化率达到 62.9%， 统筹城乡发展水平居全省首位。城乡一体的交通设施网络不断健全，实现中心城区 15 分钟上高速、主副中心城市间 30

分钟直达、与周边主要城市 60 分钟互通。五年间，生态在整治攻坚中突飞猛进。全面构建政府主导、部门联动、公众参与的环境保护治理大格局，累计消灭垃圾河 898 公里，整

治黑臭河 996 公里，新建污水收集管网 2161 公里，完成“三

改一拆”面积 1.6 亿平方米，生猪存栏量从 294.7 万头削减

到 18.3 万头，市控以上断面Ⅳ类以上水体比重从 21.9%提高到 93.1%，水环境质量从根本上实现了历史性转变。五年间， 民生在共建共享中跨越提升。城镇居民、农村居民人均可支配收入年均增长 9.4%和 9.8%，农村居民收入连续 13 年列全省第一。城乡居民养老保险、医疗保险加快从制度全覆盖迈向人群全覆盖。全市医疗卫生服务机构达到 1447 个，平均

每千人拥有医生数 2.96 名。图书馆、文化馆“总分馆”建成率 100%。五县两区全部通过国家义务教育均衡发展评估。成功蝉联全国文明城市。实现省级平安市、县（市、区）创建“满堂红”，十二年不懈奋斗，如愿获颁平安金鼎。

各位代表！过去五年，我们经受住了各种困难风险的考验，取得了改革发展稳定重大成就。这是中共嘉兴市委正确领导的结果，是全市上下齐心协力、奋力拼搏的结果。在此， 我代表市人民政府，向全市人民和广大建设者，表示衷心的感谢！向人大代表、政协委员，各民主党派、工商联、无党派人士和人民团体，向驻嘉部队、武警官兵，表示衷心的感谢！向关心支持嘉兴发展的港澳台同胞和海内外朋友们，表示衷心的感谢！

我们也清醒地看到，我市经济社会发展中还存在不少矛盾和问题，主要有：经济下行压力仍然较大，实体经济发展面临不少困难；科技创新还未成为推动发展的主动力，产业层次有待进一步提升；中心城市能级不高，集聚辐射带动能力不强；生态环境质量与群众期望仍有距离，治水、治气等

工作还需要付出更大努力；交通、教育、医疗、养老等民生工作尚有不少短板，食品安全、安全生产、社会治安等方面还存在薄弱环节。同时，一些政府工作人员不作为问题依然存在，消极腐败现象时有发生，廉政建设和反腐败斗争任务依然艰巨。我们一定高度重视这些问题，不辱使命，不负重托，勇于担当，采取更加有力措施全力以赴做好工作。

二、今后五年奋斗目标和主要任务

今后五年是嘉兴经济社会转型发展的关键时期，也是高水平全面建成小康社会的决胜阶段。市第八次党代会描绘了嘉兴未来五年发展的宏伟蓝图，提出要在高水平全面建成小康社会征程中奋勇当标尖的新使命。我们必须清醒认识和把握当前所处的历史方位，进一步坚定信心，抢抓机遇，努力谱写嘉兴科学发展、创新发展的新篇章。

今后五年政府工作的总体要求是：高举中国特色社会主义伟大旗帜，以邓小平理论、“三个代表”重要思想、科学 发展观为指导，深入贯彻习近平总书记系列重要讲话精神， 紧紧围绕“五位一体”总体布局和“四个全面”战略布局， 牢固树立以人民为中心的发展思想，积极践行新发展理念， 适应把握引领经济发展新常态，弘扬红船精神、秉持浙江精神，干在实处、走在前列、勇立潮头，勇当高水平全面建成小康社会的标尖，全力打造具有国际化品质的现代化网络型

田园城市，以优异成绩迎接建党一百周年。

今后五年，要集中在“高质量、均衡性”上重点发力， 进一步提高发展的“含金量”，增强发展的“平衡度”，朝着发展中高速、质量中高端、结构中高级的方向阔步迈进。到

2021 年，力争地区生产总值突破 5400 亿元，一般公共预算

收入突破 500 亿元；确保城乡居民收入增长与经济增长同步。做好今后五年工作，关键要在以下五方面重点引领、放

大优势。

（一）突出创新驱动，放大平台载体优势，加快推动新旧动能接续转换和产业改造升级。始终把创新作为引领发展的第一动力，全力推进“两区一城一走廊”建设，全盘构建政产学研金介用“七位一体”协同创新模式，全面打通科技强驱动产业强、实现经济强的创新经济通道，率先进入国家创新型城市行列。联动推进省全面创新改革试验区、省校合作示范区建设，重点在科技管理、科技投融资、人才发展等体制机制创新方面实现突破。立足“全域孵化”，打造以创新要素密集、功能错位发展、产业梯度布局为特征，在长三角地区具有特殊影响力和吸引力的科技企业孵化之城。规划建设沪嘉杭 G60 科创走廊，沿线集中规划布局一批重大科技基础设施、重点技术创新工程、开放型创新平台、高水平科技成果转化基地，为更高层次实现区域产业集聚互补与科技协同创新开辟新空间。大力实施“585”人才工程，建设人 才生态最优市。坚持制造业强市、服务业兴市战略不动摇，

着力提升制造业的智能化、绿色化、服务化和国际化水平， 促进生产性服务业向专业化和价值链高端延伸、生活性服务业向精细化和高品质转变。大力培育八大千亿产业，推进互联网经济强市、省军民融合产业发展示范基地建设，积极发展楼宇经济、全域旅游、海洋经济、现代都市型生态农业， 高标准建成一批特色小镇，加快形成嘉兴经济新的增长极增长带。

（二）突出内外联动，放大改革集群和接沪联杭优势， 提升经济发展的活力和市场化国际化水平。发挥市场在资源配置中的决定性作用和更好地发挥政府作用，统筹推进政府自身改革、供给侧结构性改革、资源要素差别化配置改革等重点领域改革，把国家和省赋予嘉兴改革试点数量上的优势加速转化为率先发展质量上的位势。深入实施国家“一带一路”战略，积极推动更高水平双向开放，鼓励要素有序流动、资源高效配置和市场深度融合，构建更加优良的法治化、国际化亲商、营商环境。大力引进高端外资、央企国资、品牌民资，支持外贸转型升级，促进新型加工贸易、市场采购贸易和跨境电商贸易加快发展，培育一批本土跨国公司。扎实推进省全面接轨上海示范区建设，全力打造创新政策率先接轨地、高端产业协同发展地、科创资源重点辐射地、一体化交通体系枢纽地和公共服务融合共享地。主动对接杭州都市圈发展，加快各类临杭功能平台开发，积极参与杭州城西科创大走廊、钱塘江金融港湾建设，深化浙东经济合作区共建，

在推动长三角城市群协同发展中发挥更大作用。

（三）突出全域统筹，放大城乡区域协同优势，全面提高城市承载力和竞争力。更加注重以城带乡、以乡促城，加快市域一体化发展步伐。按照“1640”“四百一千”空间布局和“一核三带三区”产业空间规划，统筹谋划全市基础设施建设、产业发展和公共服务供给，高水平建设“四网一路一空港”，实施中心城区快速路环线和放射线工程，建成启用嘉兴军民合用机场，加快构建“无缝衔接”的现代化综合交通枢纽。大力实施城市品质升级行动，联动推进中心城市、县（市）和小城市（新市镇）错位发展、协调并进，积极开展城市修补和生态修复，优化城市形态和功能，不断提升中心城市首位度，加快县域经济向城市经济转型。以打造新型智慧城市标杆市为契机，加强智慧应用体系建设，推动城乡管理由“管得着”向“管得好”迈进。

（四）突出绿色低碳，放大“三五共治”联动优势，着力打造江南水乡名典范。按照“绿水青山就是金山银山”的理念，全力推进“五水共治”“五气共治”“五废共治”。深化工业污水、生活污水、农业面源污染综合治理，实施大气污染防治“六大专项行动”，加大固体废弃物减量化、资源化、无害化处置力度。到 2021 年，在上游来水水质达标基础上，稳定实现市控以上断面Ⅲ类水为主，PM2.5 浓度持续下降，污泥、危险废弃物无害化处置率达到 95%以上。加快推进市域外配水工程（杭州方向），争取与杭州市民同步喝

上千岛湖水。加强耕地保护，落实主体功能区规划和环境功能区划，严格土地利用规划和用途管制。推进国家低碳城市试点，大力发展光伏、生物质能等绿色能源，构建以低能耗、低污染、低排放为基础的低碳经济发展模式。高标准建设千亩湖荡、千里绿道生态走廊，聚焦打造“绿城花海”的城市风貌。

（五）突出民生优先，放大公共服务供给优势，不断增强人民群众获得感。统筹重大民生政策，以标准化促进公共服务均等化，切实增加优质公共产品供给。推动实现更加充分、更高质量的就业，鼓励劳动、资本、技术、管理等生产要素按贡献参与分配，健全低收入群体增收机制，千方百计增加城乡居民收入。建立完善市域统筹、城乡一体的社会保险制度，加快实现社会保障全覆盖、更公平、可持续。发展繁荣各项社会事业，加强现代公共文化服务体系建设，优化基本医疗卫生和人口健康服务，促进教育优质均衡发展，构建以居家为基础、社区为依托、机构为补充、医养结合的养老服务格局。深化“平安嘉兴”建设，全面推进“三治”同步、“三社”联动，加快建立立体化、信息化社会治安防控 体系，打响“中国最具安全感城市”品牌。

各位代表！新旧相推，日生不滞。发展从来不会一帆风顺，实现梦想也绝非在一朝一夕。但只要我们矢志不渝、敢为人先，勠力同心、百折不挠，嘉兴勇当高水平全面建成小康社会标尖的宏伟目标就指日可待，嘉兴一定能够在未来区

域发展竞争中焕发出更加夺目的光彩！

三、2017 年主要工作

新的一年孕育新的希望，新的起点昭示新的辉煌。2017 年是新一届政府的开局之年,是实施“十三五”规划的重要 一年，也是推进供给侧结构性改革的深化之年。现阶段，我市经济企稳向好的基础正在不断巩固和夯实，但一些长期存在的结构性、素质性矛盾尚未得到根本解决。为此，我们必须认清形势，坚持稳中求进工作总基调，以提高发展质量和效益为中心，以推进供给侧结构性改革为主线，加强预期引导，深化创新驱动，聚焦改革、聚焦发展、聚焦民生，全面打好转型升级系列组合拳，促进经济平稳健康发展和社会和谐稳定。

结合我市实际，建议 2017 年全市经济社会发展主要预期目标为：地区生产总值增长 7%左右，其中市本级力争增长

7.2%；一般公共预算收入增长 6.5%，其中市本级力争增长 7% 左右；城乡居民人均可支配收入增长 7%，其中市本级力争增长 7%以上；节能减排降碳指标完成省下达的目标任务。

重点要处理好各方面关系，做好以下七方面工作。

（一）注重供需两端发力，保持经济平稳运行

深入推进“三去一降一补”。加快“退低进高”“退散 进集”，整治“低散弱”企业 5000 家以上，处置“僵尸企

业”25 家，淘汰 600 家企业落后产能。继续加大不良资产处置力度，大力发展直接融资，完善担保体系，积极防范和化解金融风险。严格落实房地产调控政策，有力保障城乡居民住房刚性需求，支持自住型和改善型购房，遏制投机性购房， 发展住房租赁市场，保持房地产市场平稳健康发展。健全涉企收费目录清单公示制度，加快建立制度化的企业减负机制。继续在公共服务、基础设施、生态环保等领域精准发力补短板。

积极扩大有效投资。实施“两个千亿”投资工程，确保

重大基础设施、重大产业项目、重点技术改造、生态环保投资增长 15%以上。高标准推进中德、中荷、中法、中日等国际产业合作园建设，强化专业招商、链式招商、以商引商， 力争实际利用外资 25 亿美元，浙商回归到位资金超过 460 亿元。深入谋划一批面向社会资本的基础设施和公共服务投资项目，推出政府与社会资本合作项目不少于 100 个。

保持外贸消费平稳增长。规范外贸综合服务平台建设，

开展平湖、海宁、桐乡省级产业集群跨境电商发展试点，扩大高新技术产品、名特优产品和服务贸易出口，加大先进技术装备进口。鼓励有条件的企业开展境外投资，支持企业瞄准先进技术、知名品牌、“专精特优”企业实施跨国并购。顺应“互联网+流通”趋势，加快推进城市商圈提档升级， 强化电子商务应用，积极培育新型消费热点，满足消费升级需求。

（二）振兴实体经济，加快产业结构调整

强化科技创新。推进省全面创新改革试验区建设，完善符合创新发展规律的政策体系和评价机制。加快秀洲国家高新区、嘉兴科技城等重大创新平台建设，力争实现省级以上高新技术园区县（市、区）全覆盖。全面实施科技企业孵化

之城三年行动计划，新增各类孵化机构 20 家、孵化面积 200 万平方米。实施科技企业“育苗造林”计划，新增高新技术企业 200 家、科技型中小企业 500 家。深化省科技金融改革创新试验区建设，积极发展创业风险投资，探索实施投贷联动，支持发展科技保险。全面落实人才新政，推进重点创新创业团队建设，加大“柔性”引才和产业人才扶持力度，新增高层次人才 100 名。

建设互联网经济强市和新型智慧城市标杆市。推进国家

信息经济试点城市和乌镇国家互联网创新发展试验区建设， 培育支持南湖智能终端、秀洲智慧物流、嘉善光通信、平湖微电机、海盐智能家居、海宁数字内容、桐乡量子通信等互联网产业集群发展，完成互联网项目投资 100 亿元，信息产业产值增长 12%。高水平承办第四届世界互联网大会·乌镇峰会。全面实施新型智慧城市标杆市建设，在城市治理、惠民服务、产业经济、宜居环境等领域开展大数据运用，启动

15 个智慧小镇、智慧街道、智慧园区、智慧社区、智慧商圈试点，建成城市大数据中心一期和城市综合运营管理中心。

打造制造业强市。深入实施《中国制造 2025 嘉兴行动

纲要》，培育壮大高端装备制造、电子信息、新材料等新兴 动能主导产业，加快推动传统动能有机更新，叠加实施“四换三名”“四加三减”，大力推行“三强一制造”，力争规上工业总产值突破 8000 亿元，新增工业机器人 1500 台、浙江

名牌 20 个。实施重点骨干企业规模和质量效益“双倍增” 计划，加快形成一批行业龙头企业和“隐形冠军”。以特色 小镇理念和方式改造提升开发区（园区），规划建设一批“园中园”“区中园”和“两创”中心。大力推进中电科嘉兴智 慧产业园、航天五院嘉兴军民融合产业发展中心建设，力争引进和培育军民融合高技术项目 20 个。

推动服务业增量提质。聚焦现代物流、科技与信息、金

融、文化旅游、健康服务等五大重点领域，加快创意设计、安全生产、检验检测等生产性服务业集聚区建设，推动阿里巴巴菜鸟城、山水六旗等重大项目取得更大进展，力争服务业增加值占地区生产总值比重提高 1.2 个百分点。创新发展绿色金融、普惠金融，支持嘉兴银行等地方金融机构发展， 加快推进南湖基金小镇、国际金融广场等平台建设。推动楼宇经济规范特色发展，培育一批总部楼、研发楼、电子商务楼等专业楼宇，新增税收超亿元楼宇 5 幢。整合优质旅游资源，大力发展全域旅游，加强旅游精品建设，打造运河国际旅游休闲城市。

深化农业供给侧结构性改革。完善生产、供销、信用“三

位一体”农民合作经济组织体系，积极扶持种粮大户、家庭

农场、专业合作社、农业龙头企业等现代农业经营主体，推动粮食生产功能区、现代农业园区向绿色高效发展转型。全面落实粮食安全责任制，确保粮食总产量稳定在 113 万吨以上。加大农业科技创新，积极推进“互联网+农业”，促进 一二三产融合发展。深入实施新一轮“强村计划”，发展壮大村级集体经济。完善农村土地所有权、承包权、经营权分置办法，不断提高农民收入，增强农业农村发展活力。

（三）深化改革开放，释放体制机制红利

全面推进“最多跑一次”改革。按照群众和企业到政府办事“最多跑一次、就近跑一次、不用跑一次”的目标，推动各级政府部门减权、放权、治权。加快实施行政审批“一

窗受理、集成服务”改革，深化相对集中行政许可权改革试点，推行“同城通办”模式，确保实现“最多跑一次”事项覆盖 80%以上。全面推行“互联网+政务服务”，建成全市统一的移动政务服务平台，实现所有投资项目网上审批、核准、备案。规范中介服务市场秩序，增强市场充分竞争，推动改革向行政审批中介服务机构深入。加快推进“区域能评、环评+区块能耗、环境标准”取代项目能评、环评。深化综合行政执法改革,全面推行“双随机、一公开”，健全完善

“部门联合、随机抽查、按标检查、一次到位”的监管机制。加快重点领域和关键环节改革。深化资源要素差别化配

置改革，制定实施统一的环境、技术、质量、安全等市场准入标准，完善企业绩效综合评价和结果运用机制，促进资源

要素向有竞争力的企业集聚。开展国家排污许可制度改革试点，建立固定污染源排污“一证式”管理制度。加快财税体制改革，重点强化预算绩效管理，稳步推进国库集中支付电子化。抓好国资国企改革，加快企业分类改革步伐，建立完善外派监事会制度，提高国有资本配置和运营效率。支持嘉善县域科学发展综合配套改革、平湖产业结构调整机制创新试点、海盐中欧城镇化伙伴关系示范区等改革试点，及时总结推广经验。

更大力度接轨上海。健全接轨上海协调推进机制。加强

交通基础设施互联互通，加快杭平申线航道、G228 国道等工程建设，做好沪乍杭铁路、通苏嘉铁路等项目前期工作， 开展沪嘉通勤快线、轨道交通对接等规划编制。加快打造长三角（沪嘉）产业协同创新区，依托平湖张江长三角科技城、漕河泾开发区（海宁分区）等共建平台，实现承接高端产业转移规模化。加大对沪招商力度，力争引进亿元以上重大产业项目 40 个。积极对接上海高端教育、医疗等公共服务资源，争取纳入上海异地双向刷卡结算首批先行地。认真落实国家区域发展战略，切实做好山海协作和援藏援疆援青等对口支援工作。

（四）推进城乡区域统筹发展，提升城市品质

加快市域一体化建设。深化“多规合一”试点，健全规划管控机制，提高市域开发建设的科学性、有序性。加快推进杭州湾跨海大桥北接线二期、杭州至海宁城际铁路等重大

交通设施建设，开展轨道交通建设规划编制，力争嘉兴军民合用机场项目可研报告通过评审。加快实施市域引水供水、污水管网、垃圾处理等城市“生命线”工程，开工建设市域外配水工程（杭州方向）。加大滨海开发，推进浙北集装箱 运输通道、黄姑塘航道改造工程建设，打通海河联运“最后一公里”，力争嘉兴港集装箱吞吐量突破 140 万标箱。

强化中心城市引领。深入实施中心城市有机更新，加快

民丰冶金片区、东栅老镇片区更新改造，开工建设子城广场、南湖湖滨公园，全面完成海绵城市建设试点任务。加大城市交通治堵力度，重点打通三环南路、三元路等断头路，科学规划建设公共停车场，进一步提升公交服务质量。启动城市快速路项目前期。健全城市管理长效机制，推动执法重心下移和执法事项属地管理，全面完成市、区两级智慧城管平台建设。高水平推进国际商务区、秀洲国家高新区、嘉兴科技城等城市重点功能区开发建设，大力发展先进制造业和高端服务业，不断增强中心城市的科技创新、金融支持、公共服务、人才集聚等功能。

补齐镇村建设短板。落实“四百一千”规划布局，完善

镇村规划体系。全面实施小城镇环境综合整治，开展道乱占治理等“六大专项行动”，加强镇区主要街道和出入口改造， 加大古建筑、老街巷等人文景观的恢复和保护力度。以景区建设的理念提升美丽乡村建设水平，扎实开展农村土地综合整治，做好村庄规划和农房设计，增强镇村配套服务功能，

打造一批美丽宜居示范村（社区）和 A 级景区村庄。

（五）狠抓环境治理和净化，打造“美丽嘉兴”

深化“五水共治”。坚持水岸同治、城乡联治、区域共 治，全力实施剿灭劣Ⅴ类水行动，加快污水输送管网和污水处理设施建设，加大规模生猪养殖场整治提升和监管力度，

健全农村生活污水处理设施长效运维机制，全面消除劣Ⅴ类水体，Ⅳ类以上水体稳定在 95%以上。大力开展河湖水生态修复、集中式饮用水水源地规范化创建，完成河道清淤疏浚

1000 万立方米。深化“河长制”，落实最严格的水资源管理制度，完善跨行政区域联合治水机制。

推进“五气共治”。部署开展机动车污染防治、城市扬

尘和烟尘整治等专项行动，完成 17 家热电企业超低排放技

术改造，淘汰燃煤小锅炉 600 台，力争全市原煤消费实现负

增长。抓好 7 个重点区域大气污染综合整治，打造一批绿色制造示范项目、示范园区，全面改善区域环境质量。探索建立市、县、镇、村四级网格化治气体系，健全公众参与机制， 提升重污染天气应急处置能力，力争市区空气质量优良率达到 75%。

实施“五废共治”。统筹推进生活垃圾、建筑垃圾、工

业固废、农业废弃物和医疗废物治理。加强固废处置能力建设，鼓励发展与循环经济相结合的资源综合利用项目。科学实施生活垃圾分类。强化固废收集、贮存、转移、处置和综合利用全过程监管，提高危险废弃物无害化处理率。

加快治危拆违。深入推进“三改一拆”，全面实施城乡

危旧房改造专项行动，重点拆除违建出租房、违建小作坊等违法建筑，做到应拆尽拆。积极开展无违建创建活动，健全违法建筑及时发现和依法处置的长效机制，力争创建无违建县（市、区）1 个，完成拆改面积 2000 万平方米以上。

（六）强化发展惠民，保障和改善民生

扩大就业完善社保。实施城乡一体的就业创业政策，重点抓好大学生红船“两创”孵化中心、农村电子商务创业小 镇等就业创业基地建设，新增城镇就业 6.6 万人。做好各类社会保险制度衔接和待遇调整，加快基本医疗保险和生育保险 整合，推动医保跨区域异地结算联网。落实支出型贫困家庭

救助政策，实现对困难家庭“一户一档一策”的精准帮扶， 确保低收入群体人均可支配收入增长 10%。创新住房保障模式，加大棚户区改造力度，新开工建设保障性住房 5748 套。加快残疾人全面小康进程，大力发展慈善事业和红十字事业。

打造文化强市。大力弘扬社会主义核心价值观，深入开

展精神文明创建活动。深化国家公共文化服务体系示范区建设，完善图书馆、文化馆“总分馆”服务模式，拓展“文化有约”平台功能，打响公共文化服务“嘉兴品牌”。启动市 文化艺术中心项目建设，完成市博物馆二期、市图书馆二期工程。大力扶持文艺创作，推出一批具有较高艺术水准和嘉兴本土特色的文艺精品。做好档案、地方志工作。加强文化遗产保护和利用，推进子城遗址考古发掘与展示工程，加快

马家浜遗址公园建设。积极引导文化消费，培育壮大文化创意、文博会展等新业态，促进文化产业创新发展。

加快教育现代化。实施新一轮学前教育行动计划，建立

学前教育“县级统筹、镇级配合”管理体制。科学实施中小学布局规划，优化城乡教育资源配置。开展高中课程改革， 提升各类特色高中办学质量。开工建设北师大嘉兴附中迁建项目。深化职业教育“产教融合、校企合作”，大力弘扬工匠精神，建设一批公共实训基地。加大对嘉兴学院等在禾高校的支持力度，深化与清华大学、同济大学、中科院等大院名校的全面合作。统筹办好特殊教育、继续教育、老年教育。健全民办学校分类登记管理制度，促进民办教育健康发展。

建设健康嘉兴。完善三级医疗卫生服务体系，深入推进

“双下沉、两提升”，积极推行分级诊疗和家庭责任医生签约制度，实现县级以上综合医院诊间结算全覆盖。大力发展中医药事业。落实全面两孩政策，优化计划生育服务管理。加快城乡居家养老服务照料中心提档升级，推进“医养结合” 试点，实施长期护理保险制度，支持社会力量兴办老年护理院、社区护理站。协调发展体育事业和体育产业，加快建设市全民体育健身中心。

创新社会治理。加强基层治理能力建设，全面建成镇（街

道）综合治理、综合执法、市场监管、便民服务“四个平台”。大力实施“七五”法治宣传教育，完善覆盖城乡的公共法律服务体系。健全新型居住证制度，引导新居民有序流动。全

面落实信访工作责任制，推动信访积案化解。加强社会信用体系建设，提高社会诚信意识。发展民族宗教、外事侨务、对台事务等事业，支持工会、共青团、妇联、科协等开展工作。加强食品药品安全监管，建立健全食品药品质量追溯制度。强化安全生产责任体系建设，加大隐患排查治理力度， 坚决遏制重特大事故发生。深化社会治安综合治理，严密防范和严厉打击各类违法犯罪活动。支持驻嘉部队和武警部队建设与改革，继续做好国防动员、国防教育、人民防空、双拥优抚、国家安全等工作。

办好民生实事。按照民生实事“群众提、委员议、代表

定、政府办”的新理念，扎实办好人代会票决的十项民生实事，着力解决人民群众普遍关心的突出问题。

（七）加强自身建设，提高政府履职水平

聚力建设法治政府。言必有主，行必有法。始终坚持法无授权不可为、法定职责必须为，把政府的一切活动纳入法治轨道。坚持依法决策、民主决策、科学决策，完善重大事

项公众参与、专家论证、合法性审查等制度。深化行政复议体制改革。健全行政权力监督和制约机制，自觉接受人大依法监督、政协民主监督和监察监督、司法监督，加强政府内部层级监督和审计监督，主动接受社会监督、舆论监督。

聚力建设服务政府。坚持问题导向，更多运用市场化、

社会化、信息化的方式加强服务管理。优化政府运行机制， 完善目标管理体系，建立重点工作责任清单制度。加强政府

信用建设，把诚信施政作为重要准则。全面推进政务公开制度化、标准化，做好信息发布和政策解读，及时回应社会关切。创新政府向社会购买服务机制。持续优化政务流程，提升便民服务效能，坚决除烦苛之弊，开便利之门。

聚力建设廉洁政府。不怕法律无情，只怕自身不清。切

实把纪律规矩挺在前面，坚持用制度管权管事管人。认真落实全面从严治党主体责任，严格履行“一岗双责”。推动中央八项规定精神风化俗成，坚决防止“四风”反弹回潮。深化廉政风险防控，强化对权力运行的监督制约，健全不敢腐、不能腐、不想腐的体制机制。坚持有腐必反、有贪必肃，坚决惩处各类腐败行为。

各位代表！新的目标激励人心，新的任务艰巨光荣，使命重在担当，实干成就辉煌。让我们在省委、省政府和中共嘉兴市委的坚强领导下，不忘初心、继续前进、勇立潮头、勇当标尖，以优异成绩迎接党的十九大胜利召开，奋力谱写高水平全面建成小康社会的崭新篇章！
